# Modeling HIV Pre-Exposure Prophylaxis

**DOI:** 10.3389/fphar.2019.01514

**Published:** 2020-01-31

**Authors:** Thomas Straubinger, Katherine Kay, Robert Bies

**Affiliations:** ^1^ Department of Pharmaceutical Sciences, State University of New York at Buffalo, Buffalo, NY, United States; ^2^ Metrum Research Group, Tariffville, CT, United States

**Keywords:** pharmacokinetics, pharmacodynamics, HIV, PrEP, Truvada, tenofovir, emtricitabine, maraviroc

## Abstract

Pre-exposure prophylaxis (PrEP) has emerged as a promising strategy for preventing the transmission of HIV. Although only one formulation is currently approved for PrEP, research into both new compounds and new delivery systems for PrEP regimens offer intriguing challenges from the perspective of pharmacokinetic and pharmacodynamic modeling. This review aims to provide an overview the current modeling landscape for HIV PrEP, focused on PK/PD and QSP models relating to antiretroviral agents. Both current PrEP treatments and new compounds that show promise as PrEP agents are highlighted, as well as models of uncommon administration routes, predictions based on models of mechanism of action and viral dynamics, and issues related to adherence to therapy. The spread of human immunodeficiency virus (HIV) remains one of the foremost global health concerns. In the absence of a vaccine, other prophylactic strategies have been developed to prevent HIV transmission. One approach, known as pre-exposure prophylaxis (PrEP), allows HIV-negative individuals who are at high risk of exposure to the virus, be it through an HIV-positive sexual partner or through the shared use of drug injection equipment, to substantially reduce the risk of developing an HIV infection. PrEP is a relatively recent approach to combating the HIV epidemic, with the only currently approved treatment being Truvada, a daily oral antiretroviral (ARV) therapy initially indicated in the treatment of active HIV-1 infections, but approved for HIV PrEP in 2012. Although PrEP therapy has consistently demonstrated high efficacy in preventing HIV infection, this efficacy is dependent on patient adherence to the prescribed treatment regimen. This can present a significant problem in low- and middle-income countries, which may lack the infrastructure to provide sufficient access to PrEP medication to maintain daily dosing regimens. Furthermore, while the conventional approach has generally been to advocate for continuous administration akin to regimens used for viral suppression in infected patients, there has been some discussion of whether a better treatment paradigm might be to push for PrEP therapy primarily during those known periods of heightened exposure risk, while relying on post-exposure prophylaxis regimens to prevent infection after unanticipated exposures during low-risk periods. These considerations have led to a push for the development of long-duration and on-demand PrEP formulations, including subdermal and subcutaneous implants, slow-release intramuscular depot injections, vaginal and rectal antimicrobial gels, and intravaginal rings and dissolving films. PrEP therapy is a quickly evolving field, with a variety of antiretroviral compounds and formulations under investigation. This review aims to report on notable drugs and formulations from a pharmacokinetic/pharmacodynamic (PK/PD) modeling perspective. Given the nature of PrEP as a preventive therapy designed for long-term use, clinical trials for PrEP therapies can last for months or even years, particularly in the case of long-duration formulations. Furthermore, in contrast to antiretroviral trials in infected patients, pharmacodynamic endpoints in PrEP therapies are difficult to quantify, as the primary endpoint for efficacy is generally the rate of seroconversion. Computational modeling approaches offer flexible and powerful tools to provide insight into drug behavior in clinical settings, and can ultimately reduce the time, expense, and patient burden incurred in the development of PrEP therapies.

## Current and Potential Prep Therapies

### Tenofovir Disoproxil

Tenofovir (TFV) is a nucleotide reverse transcriptase inhibitor (NRTI), a nucleoside phosphonate analogue of the endogenous nucleoside monophosphate, or nucleotide, adenosine 5’-monophosphate, and was one of the first compounds identified as a potential candidate for HIV prophylaxis. A 1995 study demonstrated that subcutaneous injections of TFV could protect macaques from simian immunodeficiency virus (SIV). (Tsai et al., 1995; Kearney et al., 2004) Tenofovir disoproxil fumarate (TDF) is a prodrug of TFV and has been in use for HIV treatment in the US since 2001. (Chapman et al., 2003) Studies have demonstrated the efficacy of TDF with and without emtricitabine (FTC) in preventing HIV infection in a variety of populations, including men who have sex with men (MSM), transgender women, heterosexual men and women, and people who inject drugs. (Grant et al., 2010; Baeten et al., 2012; Thigpen et al., 2012; Choopanya et al., 2013) Two major studies were terminated due to a lack of efficacy, however in both studies blood samples revealed that despite high self-reported adherence rates among patients in the treatment arms, actual adherence rates were low, with the fraction of patients with detectable plasma levels of drug ranging from 23-40%. (Van Damme et al., 2012; Marrazzo et al., 2015)

Preclinical testing revealed that TFV has low oral bioavailability due primarily to the ionic charges on its phosphonate group. (Cundy et al., 1998) The structure of TDF masks these charges, improving intestinal absorption and making an oral formulation feasible. (Shaw et al., 1997) After absorption in the intestine, TDF is converted into TFV through hydrolysis of its two ester groups. TFV is therefore the primary circulating compound in TDF-based treatments. (Kearney et al., 2004)

After uptake into cells, TFV undergoes sequential phosphorylation by adenylate kinase and nucleoside diphosphate kinase into its active form, tenofovir diphosphate (TFV-DP). TFV-DP inhibits HIV-1 replication by competing with endogenous deoxyadenosine 5’-triphosphate (dATP), inhibiting HIV-1 activity and halting strand elongation when incorporated into viral DNA.

Several pharmacokinetic models of TFV have been developed, but relatively few have focused specifically on PrEP therapy. Duwal et al. developed a pharmacokinetic model linking plasma concentrations of orally-administered TDF to intracellular concentrations of TFV-DP, which is used to drive a viral dynamics model. (Duwal et al., 2012) This model allows for the estimation of prophylactic efficacy while taking into account variable dosing of TDF, a necessity given that variability in adherence to the prescribed dosing regimen has been observed as a determinant of the efficacy of PrEP therapy. A two-compartment model was used to describe the PK of TFV, with a first-order rate constant describing the absorption of TDF and its conversion to TFV. A third compartment is used to depict the intracellular concentration of TFV-DP, with a Vmax model describing the saturable processes of cellular uptake of TFV and its phosphorylation to TFV-DP. A diagram of the compartmental model is included in **Supplementary Figure 1**. The group chose to ignore inter-individual variability in the plasma pharmacokinetics of TFV, as it is arguably negligible relative to the degree of variability in the intracellular pharmacokinetics of TFV-DP. The pharmacodynamic model borrowed a hybrid stochastic-deterministic model of viral dynamics described by von Kleist et al. (von Kleist et al., 2011) Briefly, the model incorporated free infectious and non-infectious virus, as well as uninfected, early infection, and late-stage infection T-cells and macrophages. For each possible event in the infection process, such as infection of a cell, integration of the viral genome, or the production of new virus particles, the rate constant is determined by both the quantity of the species involved and a propensity function describing the likelihood of the event occurring. If either the propensity function or the quantity of any of the species involved in a given reaction are below a pre-specified threshold, that reaction is modeled as a stochastic process. Otherwise each reaction is treated as a deterministic process. Simulations of HIV challenges suggested that variability in adherence had little effect on the efficacy of TDF PrEP therapy for adherence above 60%, but the effect became significant when adherence dropped below 40%. However, the size of the viral inoculum had a significant impact on efficacy regardless of adherence rates. This leads von Kleist et al. to suggest that TDF-based PrEP may be most effective when used in the prevention of sexual transmission of HIV, as this route generally involves smaller inoculum sizes than transmission *via* shared needles or blood transfusions.

Prophylactic therapies against HIV require sufficient drug concentrations at the site of exposure. As sexual contact is the most common route of transmission, characterizing the distribution of antiretrovirals in anogenital tissues is of particular importance in the development of HIV PrEP therapies. (Centers for Disease Control and Prevention, 2018) Collins et al. recently published a population PK model relating plasma and rectal tissue concentrations of TFV, demonstrating that non-linear mixed-effects (NLME) modeling is a viable approach for predicting TFV tissue exposures using a sparse tissue and rich plasma sampling scheme. (Collins et al., 2017) A diagram of the compartmental model used by Collins et al. can be found in **Supplementary Figure 2**.

Various long-duration formulations of TFV are being investigated for PrEP. Vaginal gel, ring, and film formulations have been developed with the goal of providing women in high-risk populations with multiple options for prophylaxis in an effort to improve adherence. More recently, there have been efforts to develop rectal topical TFV formulations, as receptive anal intercourse is a common route of exposure to HIV.

Gao and Katz created a multicompartment physiological model for the pharmacokinetics of TFV administered *via* a vaginal gel. (Gao and Katz, 2013) The model allows for the simulation of concentrations across the vaginal mucosa, with dedicated compartments for the gel, vaginal epithelium, stroma, and uptake into the blood and lymphatic systems. This model offered insights into the spatial distribution of TFV throughout the layers of the vaginal mucosa, which is important for assessing whether prophylactic concentrations of TFV are being achieved in the vaginal stroma. Additionally, it suggested that variations over the course of a menstrual cycle, such as changes in the thickness of the epithelium, could have a significant impact TFV transport into the stroma.

More recently, Gao and Katz published a physiological model for TFV administration *via* an enema delivery vehicle. (Gao and Katz, 2017) Compared to the vaginal delivery model, the geometry of the colorectal canal is fairly complex, with both macroscopic folds and creases and microscopic, columnar, fluid-filled crypts in the rectal wall. As a result, modeling rectal drug delivery requires a more detailed mathematical description of the movement of the delivery vehicle itself. Given the larger overall surface area and thinner epithelium of the rectal mucosa, the model predicts much more rapid delivery of TFV *via* rectal administration than *via* vaginal.

An important aspect of PK/PD studies of topically-administered microbicides is accurately and reliably characterizing drug concentration profiles in tissues. This can be difficult due to both inherent variabilities in drug concentrations in mucosal tissues and luminal fluid, and limitations in the frequency with which tissue biopsies can be performed. In contrast, acquiring pharmacokinetic data from blood samples is relatively simple and can be carried out more frequently to provide a richer depiction of the pharmacokinetic profile than might be possible from fluid or tissue samples. Recently, Govil and Katz published a proof of concept study of a modelling approach utilizing feedforward neural networks to link plasma pharmacokinetic models of TFV to vaginal tissue PK and PD endpoints. (Govil and Katz, 2019)

### Emtricitabine

Emtricitabine (FTC) is a nucleoside reverse transcriptase inhibitor effective against HIV-1. In the context of PrEP, FTC is administered as a combination oral therapy with the NRTI tenofovir disoproxil fumarate. Like tenofovir, FTC undergoes intracellular phosphorylation to its active form, emtricitabine 5’-triphosphate (FTC-TP), an analogue of deoxycytidine 5’-triphosphate (dCTP). Incorporation of FTC-TP into HIV-1 DNA during viral DNA replication terminates chain elongation. (Modrzejewski and Herman, 2004)

A recent model published by Garrett et al. found that FTC plasma concentrations were best described by a two-compartment PK model with first-order absorption and saturable metabolite formation, similar to the previously described model for TDF. (Garrett et al., 2018) The metabolite FTC-TP is described by a one-compartment model representing concentration within peripheral blood monocytes (PBMCs), the main site of action, with movement from the intracellular space to plasma represented by a first-order process.

FTC has not been investigated as a monotherapy for HIV PrEP. However, Valade et al. have published a population model for FTC in HIV-1 infected patients with varying degrees of renal impairment, as renal elimination appears to be a primary determinant of FTC pharmacokinetics. (Valade et al., 2014) This model was later expanded to include seminal plasma FTC concentrations in MSM, as a measure of both viral suppression and to characterize concentrations in male genital tissues. (Valade et al., 2015) The parameter estimates from these models are shown in **Table 1**. In addition, non-compartmental PK parameters for FTC are included in **Supplementary Table 1**. Although not necessarily directly applicable to PrEP therapies, these models may provide initial values for future models of FTC.

**Table 1:** Model-Specific Values for Emtricitabine^a^.

Parameter	Value in Male and Female Adult Patients (%RSE)	Value in MSM (%RSE)
CL/F (L/h)	15.1 (6)	14.8 (4)
V_c_/F (L)	42.3 (12)	51.6 (11)
Q/F (L/h)	5.75 (38)	8.19 (26)
V_p_/F (L)	55.4 (28)	106 (44)
k_a_ (h^-1^)	0.53 (Fixed)	0.53 (Fixed)
Effect of creatinine clearance on CL/F	0.278 (23)	0.178 (35)
Inter-Individual Variability of CL/F	0.174 (14)	0.255 (12)
Residual Variability (Proportional Error)	0.422 (7)	0.339 (6)

^a^Parameter values taken from (Valade et al., 2014; Valade et al., 2015).

### Tenofovir Disoproxil and Emtricitabine

Originally approved in 2004 for the treatment of HIV infection, a fixed-dose, oral, combination TDF-FTC therapy, Truvada received approval in 2012 for use as a PrEP therapy in individuals at high risk of contracting HIV and was the first therapy approved for HIV PrEP. (U.S. Food and Drug Administration, 2012) The use of a combination therapy incorporating two different nucleotide analogues provides a synergistic effect and reduces the impact of resistance to either of the two drugs individually. Additionally, the incorporation of nucleoside analogues during reverse transcription is a saturable process. Each viral DNA sequence contains a finite number of each nucleoside, so by targeting multiple nucleoside, the overall probability of incorporating an inhibitory nucleoside analog is increased.

Although the pharmacokinetics of the two drugs can be modeled independently, a model published by Cottrell et al. attempts to capture the distribution of both TDF and FTC in vaginal, cervical, and rectal tissue in order to connect tissue concentrations to protective effect against HIV infection. (Cottrell et al., 2016) A diagram of the model can be found in **Supplementary Figure 3**. Their study suggested that TFV has a propensity to distribute to colorectal tissue while FTC is more prone to accumulate in the female genital tract. Furthermore, by including endogenous nucleotide concentrations, the ratios of TFV-DP to dATP and FTC-TP to dCTP can be used as PD endpoints. The distribution of endogenous nucleotides also shows tissue specificity, with significantly higher nucleotide concentrations in female genital tract tissues. Based on these tissue distribution characteristics, it was predicted that adherence to 2 of 7 weekly doses of oral TDF with or without FTC was sufficient to provide protection in colorectal tissue, while adherence to a minimum of 6 out of 7 weekly doses was necessary to protect the female genital tract from HIV infection. These predictions are consistent with the results of the iPrEX trial, in which two doses of TDF-FTC per week were sufficient to significantly decrease the risk of rectal HIV acquisition in MSM, as well as the FEM-PrEP and VOICE studies, which found that similarly low levels of adherence did not confer any reduction in the rate of vaginal HIV acquisition. (Van Damme et al., 2012; Grant et al., 2014; Marrazzo et al., 2015)

### Tenofovir Alafenamide

Tenofovir alafenamide fumarate (TAF) is a novel prodrug of tenofovir, and shows potential as areplacement for TDF in PrEP therapy. (De Clercq, 2016) In October 2019, a combination therapy of TAF and FTC became the second approved PrEP medication in the US, though it was only approved for use in men and transgender women. While TDF is an ester prodrug that undergoes rapid metabolism in plasma to TFV, TAF is primarily metabolized intracellularly by the enzyme cathepsin A. (Birkus et al., 2007) In clinical studies TAF been shown dramatically increase TFV-DP exposure in PBMCs, with a 8 mg of TAF being approximately equivalent to a 300 mg dose of TDF. (Ruane et al., 2013) An overview of the parameters of TAF and TDF is presented in **Table 2**. The fact that TAF is metabolized intracellularly reduces systemic concentrations of TFV. Unlike TFV, TAF is not a substrate for the renal organic anion transporters OAT1 and OAT3 which both reduces its rate of renal elimination and the risk of nephrotoxicity associated with TFV. (De Clercq, 2018) However, a recent meta-analysis of clinical trials comparing the efficacy and safety of TAF and TDF monotherapies with and without the pharmacokinetic enhancers ritonavir (RTV) and cobicistat (COBI) found that TAF reduced the incidence of bone mineral density depletion and had slightly better viral suppression than TDF, but only when administered with RTV and COBI. (Hill et al., 2018).

**Table 2:** Comparison of TAF and TDF Pharmacokinetics^a^.

Parameter	TAF			TDF
	8 mg	25 mg	40 mg	300 mg
TAF Multiple Dose PK, Day 10				
AUC_last_ (h ng/mL), mean (CV%)	54.7 (92.6)	115.2 (33.4)	308.9 (33.6)	
C_max_ (ng/mL), mean (CV%)	85.8 (116.3)	223.6 (58.8)	629.5 (57.0)	
T_max_ (h), median (Q1, Q3)	0.50 (0.50, 0.50)	0.50 (0.50, 0.75)	0.50 (0.38, 0.50)	
t_1/2_ (h), median (Q1, Q3)	0.38 (0.26, 0.50)	0.39 (0.34, 0.54)	0.42 (0.32, 0.49)	
TFV Multiple Dose PK, Day 10				
AUC_tau_ (h ng/mL), mean (CV%)	65.5 (23.5)	267.7 (26.7)	405.8 (12.7)	1918.0 (39.4)
C_max_ (ng/mL), mean (CV%)	4.2 (24.7)	15.7 (22.1)	28.3 (8.7)	252.1 (36.6)
C_tau_ (ng/mL), mean (CV%)	2.1 (33.8)	9.2 (26.1)	13.3 (16.0)	38.7 (44.7)
T_max_ (h), median (Q1, Q3)	1.50 (1.00, 1.98)	1.50 (1.25, 1.75)	1.29 (1.04, 1.50)	1.25 (0.58, 2.00)
t_1/2_ (h), median (Q1, Q3)	30.77 (26.90, 55.61)	40.19 (28.98, 44.84)	35.95 (26.38, 42.90)	14.86 (12.18, 16.81)
TFV-DP in PBMC, Multiple Dose PK, Day 10				
AUC_tau_ (µM h), mean (CV%)	3.5 (77.1)	21.4 (76.9)	74.5 (92.7)	3.0 (119.6)

^a^Table reproduced from (Ruane et al., 2013).

In addition to TDF and FTC, Garrett et al. included a model of TAF in their 2018 publication. (Garrett et al., 2018) Unlike TDF and FTC, they depict TAF using a single plasma compartment, likely owing to the fact that the TAF prodrug is metabolized to TFV intracellularly, drastically reducing the circulating concentrations of TFV, which is usually modeled with two-compartment disposition. A transit compartment and first order input are used to model the uptake into PBMCs, conversion into TFV, and subsequent phosphorylation into TFV-DP. Elimination from PBMCs is described as a first-order process.

### Maraviroc

Maraviroc (MVC) is a small-molecule antagonist of the chemokine co-receptor CCR5. (Dorr et al., 2005) HIV-1 infection begins with a gp120 glycoprotein trimer on the virion binding to three CD4 proteins on the target cell. This causes a conformational change in gp120 that exposes additional binding sites that must interact with a co-receptor on the cell surface, with CXCR4 and CCR5 being the two primary co-receptors used by HIV-1. Interaction with the correct co-receptor allows a second protein, gp41, to undergo a conformational change and penetrate the cell membrane of the target cell, which in turn allows membrane fusion between the HIV-1 virion and target cell, followed by the release of HIV-1 RNA into the cytoplasm of the host cell. (Panos and Watson, 2015) HIV-1 strains can display an affinity, or tropism, toward utilizing either CXCR4 or CCR5, in which case they are referred to as the X4 or R5 variants, respectively. Interestingly, the relative prevalence of these variants shifts over the course of the disease, with the R5 variant being far more prevalent during the initial infection, with the X4 variant gradually increasing as the disease progresses toward AIDS. (Berger et al., 1999) The reason for this shift in tropism has not been definitively established, but what is clear is that the R5 variant plays a key role in HIV-1 transmission, so much so that two individuals with homozygous mutations in the CCR5 gene proved extremely resistant to HIV-1 infection, despite repeated exposures. (Liu et al., 1996) This makes CCR5 an attractive target for PrEP therapy, as it appears to be integral to the establishment of the initial HIV infection.

Dose escalation studies of MVC in healthy volunteers found it was well absorbed after oral absorption, reaching T_max_ within 30 min to 4 h post dose. (Abel et al., 2008b) MVC exhibits non-dose proportional pharmacokinetics, with higher dose levels leading to proportionally smaller increases in AUC and C_max_. The absolute oral bioavailability of MVC was estimated at 23% for an oral dose of 100 mg, increasing to 33% for a dose of 300 mg. (Abel et al., 2008a) Mass-balance analysis suggested 60% of orally administered MVC is lost to first pass metabolism. (Abel et al., 2009) MVC is a substrate for both the metabolizing enzyme cytochrome P450 (CYP) 3A4 and the efflux transporter P-glycoprotein, which likely accounts for the non-proportional pharmacokinetics. (Abel et al., 2001) Approximately 23% of MVC clearance is renal, the remaining 77% is believed to be metabolic, and overall clearance does not appear to be affected by dose. (Abel et al., 2008a). A table of non-compartmental parameters from the FDA clinical pharmacology and biopharmaceutics review of MVC can be found in **Supplementary Table 2**.

Chan et al. developed a population pharmacokinetic model of MVC based on a meta-analysis of 17 phase 1 and 2 studies in both healthy and HIV-infected subjects, and the resulting parameter estimates are presented in **Table 3**, and a diagram of the model can be found in **Supplementary Figure 4**. (Chan et al., 2008) MVC disposition was characterized by a two-compartment model, with drug input from oral dosing described by a first-order absorption rate constant with a time delay. The model incorporated a sigmoidal E_max_ model to describe the nonlinearity of the extent of absorption (F_abs_), with F_abs_ expressed as a function of ABS_Emax_, the maximum fraction absorbed, and ED50, the dose producing 50% of maximal absorption. A power function was used to describe the relationship between the absorption rate constant (ka) and dose. The effect of food emerged as a significant covariate, with a fed state causing a linear reduction in ka and an exponential reduction in ABS_Emax_ and ED50. Interpatient variability was included on ED50, hepatic extraction ratio (E_H_), intercompartmental clearance (CL_ic_), absorption rate constant, and central and peripheral volumes of distribution (V_c_ and V_p_). Both race and age also emerged as statistically significant covariates, with race affecting V_p_ and CL_ic_ and age influencing CL_ic_. In the final model, race was implemented as a binary variable of Asian vs. non-Asian. In Asian subjects, estimates for E_H_ were reduced by approximately 14%, which translated into a 17.7% increase in F due to a reduction in first-pass hepatic elimination. Asian patients were also estimated to have a 1.8% decrease in CL_ic_ and only a 0.23% decrease in V_p_. Age emerged as a covariate for CL_ic_, with an increase of 0.349 L/h for each year of age over 30. Despite being statistically significant, the differences due to race and age were deemed to be clinically insignificant, requiring no dose adjustment. Weight, sex, and HIV status were also included in the covariate modeling process, but had no significant impact on model parameters. The majority of residual error occurred during the absorption phase, so the error model was fit as a function of time after dose.

**Table 3:** Model-Specific PK Parameters for Maraviroc^a^.

Parameter	Description	Final Model Estimate (%SE)	1000 Bootstrap Run Statistics Mean ( ± SD)	CV%
Sturctural Model				
CL (L/h)	Systemic clearance	51.45^b^		
E_H_	Hepatic extraction ratio	0.662 (1.5)	0.66 (0.01)	1.52
E_H,__Race_	Effect of race on E_H_	-0.0948 (15.0)	-0.09 (0.01)	13.77
V_c_ (L)	Central compartment volume	132 (2.7)	131.6 (3.08)	2.34
CL_ic_ (L/h)	Inter-compartment clearance	16.4 (3.9)	16.27 (0.57)	3.52
CL_ic,_ _Race_	Effect of race on CL_ic_	-0.298 (17.7)	-0.29 (0.05)	15.82
CL_ic,_ _Age_	Effect of age on CL_ic_	0.349 (28.3)	0.35 (0.09)	26.21
V_p_ (L)	Peripheral compartment volume	277 (4.2)	276.07 (10.13)	3.67
V_p,__Race_	Effect of race on V_p_	-0.637 (8.5)	-0.63 (0.05)	7.35
k_a_ (h^-1^)	Absorption rate constant	0.277 (21.0)	0.28 (0.06)	20.64
θ_ka_	Exponential change in k_a_ relative to 1 mg dose	0.173 (22.9)	0.18 (0.04)	22.82
θ_ka,__1__mg_	Fed-state fractional change in fasted k_a_	0.547 (18.6)	0.56 (0.10)	17.95
ABS_Emax_	Maximum fraction absorbed	1 (Fixed)		
θ_ABS_ _Emax_	Fed-state exponential change in E_max_	-0.258 (27.3)	-0.24 (0.09)	36.23
ED_50_	Dose producing half-maximal absorption	51.2 (12.8)	50.82 (8.06)	15.87
θ_ED50_	Fed-state exponential change in ED_50_	0.594 (34.5)	0.66 (0.41)	62.46
γ	Sigmoidicity	1.39 (15.3)	1.38 (0.30)	21.9
T_lag_ (h)	Absorption Lag time	0.198 (4.1)	0.20 (0.01)	4.13
FQ (L/h)	Hepatic blood flow	59.59 (Fixed)		
Residual Error				
P_max_ (%)	Maximum change in residual variability from baseline	74.2 (3.9)	74.59 (2.43)	3.26
T_max_ (h)	Time of maximum residual variability	0.950 (8.8)	0.95 (0.08)	8.35
K	Exponential rate constant for residual variability	0.403 (6.4)	0.40 (0.02)	5.99
Base (%)	Baseline residual variability	20.2 (3.3)	20.0 (0.66)	3.28
Inter-subject Variability				
ω[ED_50_] (%)	ISV of ED_50_	58.9 (32.6)	61.17 (15.03)	24.57
ω[E_H_] (%)	ISV of E_H_	8.3 (11.0)	8.27 (0.43)	5.22
ω[V_c_] (%)	ISV of V_c_	11.5 (77.3)	8.92 (5.64)	63.21
ω[CL_ic_] (%)	ISV of CL_ic_	30.5 (22.7)	29.8 (3.41)	11.45
ω[k_a_] (%)	ISV of k_a_	40.0 (13.9)	39.92 (2.59)	6.48
ω[V_p_] (%)	ISV of V_p_	27.8 (21.2)	27.23 (2.89)	10.63

^a^Table reproduced from (Chan et al., 2008).

^b^Calculated from CL_H_ + CL_R_, where CL_H_ = FQ∙E_H_, and CL_R_ is fixed to 12 L/h.

### Dapivirine

Dapivirine (DPV) is a second-generation non-nucleoside reverse transcriptase inhibitor (NNRTI). Initially intended for use in highly active antiretroviral therapy (HAART) against HIV strains resistant to first generation NNRTIs, evidence of poor oral absorption early in development led to the investigation of DPV as a topical microbicide. (de Béthune, 2010) A monthly intravaginal DPV ring under development by the International Partnership for Microbicides (IPM), which currently holds exclusive rights to DPV, has multiple formulations in development, including vaginal and rectal gels, intravaginal films, and intravaginal rings. A monthly DPV intravaginal ring being developed by IPM has been through Phase III and Phase IIIb testing, and a regulatory decision is anticipated at some point in 2019. (Baeten et al., 2016; Nel et al., 2016; Baeten et al., 2018; Nel et al., 2018)

Given the poor performance of DPV as an oral PrEP compound, and its repurposing for topical delivery, there have been relatively few modeling studies performed. Hawles et al. developed a pharmacokinetic model for intravaginal delivery *via* a DPV gel, based on the TFV gel model developed by Gao and Katz. (Gao and Katz, 2013; Halwes et al., 2016) More recently, Kay et al. published a physiologically-based pharmacokinetic model DPV delivered *via* either an intravaginal ring or film. (Kay et al., 2018b) The model captures physiological determinants of DPV absorption in the cervicovaginal tract, including compartments for the device itself, vaginal luminal fluid, vaginal epithelium, vaginal stromal tissue, and stromal blood. A diagram of the model is available in **Supplementary Figure 5**. This level of granularity in depicting the characteristics of individual tissue types is necessary given the level of variability in vaginal drug delivery.

### Long-Acting Injectable Formulations: Rilpivirine and Cabotegravir

Long-acting injectable (LAI) formulations have recently been the subject of research interest for PrEP therapy. This administration route avoids the problems with topical or enteral absorption, while allowing for long-term sustained release of drug into systemic circulation. The drugs rilpivirine (RPV) and cabotegravir (CAB) have recently shown promise as a combination LAI treatment for HIV-1 infected adults. (Spreen et al., 2013)

Like dapivirine, rilpivirine is a second-generation NNRTI currently being investigated for the treatment of HIV variants resistant to common NNRTIs such as efavirenz (EFV) and nevirapine (NVP). (Ripamonti et al., 2014) Currently prescribed as an oral formulation for treatment-naïve HIV-1 patients, RPV is being investigated as a long-acting intramuscular injectable for HIV PrEP. Cabotegravir is an integrase strand transfer inhibitor (INSTI) being investigated for use in both HIV treatment and prophylaxis. Although an oral formulation is being tested, the low solubility and slow metabolism of CAB make it suitable for use as a long-acting injectable (LAI). (Cattaneo and Gervasoni, 2018) To date, CAB and RPV have undergone separate clinical trials for HIV PrEP in the ECLAIR and MWRI-01 studies, respectively. (McGowan et al., 2016; Markowitz et al., 2017) Clinical trials assessing CAB and RPV combination LAI formulations have yet to be undertaken.

Rajoli et al. developed a general physiologically-based PK model for LAI formulations. (Rajoli et al., 2015) Although the model was initially validated using oral drug formulations, it was able to simulate the pharmacokinetics of LA RPV administered *via* intramuscular injection. Unfortunately, the model does not include tissue compartments that are relevant to PrEP, such as rectal and female genital tract tissues. Despite this, it may serve as a useful starting point for future physiologically-based models of LAI formulations.

## Viral Dynamics and Pharmacology

Ultimately the goal of PK/PD modeling is to connect drug exposure to clinical response. In the case of modeling antiretroviral therapies for HIV, this requires some description of HIV viral dynamics. Pharmacodynamic parameters can be derived from *in vitro* and ex vivo assays, but caution must be exercised when attempting to translate these results to *in vivo* efficacy. Tissue explant models, for example, can demonstrate high levels of inter-patient variability in infectivity. (Kay et al., 2018a) Furthermore, a large viral inoculum is required to establish an infection in ex vivo systems, far in excess of what would be required *in vivo*. While HIV dynamics in an active infection can generally be modeled as a deterministic process, the underlying behavior of individual virions is inherently stochastic. A very small number of initial virions serve as progenitors during the initial infection, which is best described as a stochastic process. (Carlson et al., 2014)

Duwal et al. have described a multiscale modeling approach for predicting the efficacy of HIV PrEP candidates. (Duwal et al., 2016) Their modular framework incorporates models for pharmacokinetics, viral transmission, and long term efficacy, but key to the estimation of efficacy are the viral replication and molecular mechanism of action (MMOA) models. The MMOA model was developed by Von Kleist et al, and attempts to mechanistically describe the mechanism of action of NRTIs. (von Kleist et al., 2012) Briefly, the model depicts the process of DNA polymerization using a Markov jump process, where each state in the model represents the incorporation of an additional nucleoside. From each state, the chain can either shorten through pyrophosphorolysis, extend by incorporation of a nucleoside through polymerization, or be terminated *via* incorporation of a nucleoside analog. The reaction rates for each of these process are specific to the each nucleoside and nucleoside analog. Nucleoside analogs achieve inhibition of viral replication by increasing the amount of time required to complete polymerization of viral DNA, as sequences incorporating a nucleoside analog cannot continue the polymerization process until the analog has been removed. If the virus cannot replicate its DNA quickly enough, it is cleared intracellularly. By computing the mean time to complete the polymerization of the full viral DNA sequence and comparing it to the mean time required for intracellular clearance, it is possible to estimate the probability of a virus successfully replicating itself. Based on the binding affinity and polymerization rate constant of both endogenous nucleosides and their analogs, it is then possible to estimate the effect of a given concentration of nucleoside analog on viral proliferation.

The effects of NRTIs on viral replication are then incorporated into a model of HIV viral dynamics. This model represents the process of infection by describing the viral replication cycle as a Markov jump process with five possible states: free virus, early infected T-cell, late infected T-cell, infected T-cell producing viral progeny, and virus cleared from the system before reaching the productive infection state. The effects of NRTIs are incorporated into the model in two ways. First, they reduce the rate of transition from the free-virus state to the early infected T-cell state, by increasing the time required for the virus to enter the cell and successfully transcribe its genome. Second, they increase the rate of clearance of the virus due to failed attempts to infect a cell. Though the study focused on the effects of NRTIs, the viral dynamics model can easily incorporate the mechanisms of other classes of antiretroviral compounds. (Duwal et al., 2019) The effects of co-receptor antagonists can be modeled as inhibition of transition from the free virus to early infection as well as inhibition of clearance due to a failed attempted infection, integrase inhibitors can be described by inhibiting the rate of transition from early to late stage infected T-cells, and protease inhibitors can impede the transition from productive infected cells to free virus.

The primary goal of this level of mechanistic detail is the prediction and identification of compounds likely to be well-suited to PrEP. The widespread use of pharmacokinetic modeling has significantly reduced the rates of drug failures due to pharmacokinetics in the later stages of development, as compounds with poor PK properties are relatively easy to screen for. Screening compounds based on their pharmacodynamics is significantly more involved, particularly in a paradigm like PrEP, where adherence and transmission rates can have a significant impact on efficacy. In their analyses, Duwal et al. identified several antiretrovirals that appear to have favorable pharmacodynamic properties. Efavirenz, nevirapine, etravirine, and rilpivirine were all found to be highly potent PrEP agents, with prophylactic efficacy maintained even after a three-day gap in administration. The group also found that maraviroc and rilpivirine maintain 50% and 72% efficacy, respectively, at low concentrations, and noted that simulations suggested that after three days of missed doses, the efficacies of raltegravir and maraviroc dropped to 8% and 50%, respectively, while rilpivirine maintained 100% prophylactic efficacy.

## Adherence and Transmission

Given that the efficacy of PrEP is highly dependent on patient adherence, it may be important to incorporate models of adherence when modeling PrEP at a population level. (Haberer et al., 2015; Fonner et al., 2016) To date, there are few published models describing HIV transmission in a population utilizing PrEP, and of those very few incorporate PK/PD. One exception is the previously described PK/PD model of FTC, TDF, and TAF created by Garrett et al., based on earlier studies by Cottrell et al. (Cottrell et al., 2016; Cottrell et al., 2017; Garrett et al., 2018) Using Monte Carlo simulations of 1000 patients each, a variety of treatment scenarios were investigated. In addition to the standard treatment doses (300 mg TDF, 200 mg FTC, or 25 mg TAF), dosing regimens included double the standard dose, steady-state dosing with one to seven doses per week, and on-demand dosing involving a double dose either 2 or 24 h pre-exposure, followed by standard treatment doses at 24 and 48 h post-exposure. All three monotherapies and both TDF + FTC and TAF + FTC combination therapies were simulated for all dosing scenarios, with protective effect estimated based on the ratio of endogenous nucleosides to nucleoside analogues. However this assumption has been criticized for failing to account for the nonlinear, saturable nature of the polymerization process. (Duwal et al., 2016)

A second notable example of a model incorporating transmission, adherence, and PK/PD is the previously mentioned multiscale modeling framework described by Duwal et al. (Duwal et al., 2016) The group incorporated a model of viral exposure to quantify the relationship between donor viral load and the number of transmitted viral particles. Briefly, they assumed a linear relationship between the log of the viral load in the donor and the log of the probability of infection, which lead to the derivation of a power function relating viral load to the number of transmitted viral particles per sexual encounter. The viral content of infected individuals was assumed to be log-normally distributed, based on observed data from individuals shortly following seroconversion. By combining estimates of viral load, the corresponding estimate of number of transmitted proteins, and the estimates from the viral dynamics model described in the previous section, an overall per-encounter probability of infection can be calculated. Finally, a population-level model incorporating the number of infected individuals and the probability of unprotected sex acts can be used with the outputs of the viral exposure model to simulate clinical trials and estimate an overall trial efficacy.

The majority of models of adherence in HIV PrEP therapy are epidemiological models of HIV transmission in a population. Although these models generally do not incorporate pharmacokinetics or pharmacodynamics, they may be informative to population PK modelers looking to capture the effects of non-adherence. One major caveat to the use of these models is their potential lack of generalizability, as the behavioral and societal factors influencing adherence rates vary with geography and culture. Even within the same geographic region, different subpopulations may exhibit different rates of adherence to PrEP, which may make it difficult to develop a generalized model of adherence.

A 2008 paper by Vissers et al. details a simulation study of various PrEP therapy scenarios in Botswana, Nyanza Province in Kenya, and Southern India. (Vissers et al., 2008) This study focuses on HIV transmission in the sex industry, with sex workers and their clients considered high risk relative to the rest of the population. The group used a compartmental model adapted from earlier models of antiretroviral therapy and male circumcision interventions.(Nagelkerke et al., 2002; Nagelkerke et al., 2007) Briefly, the model population is stratified high- and low-risk groups, with compartments for uninfected, uninfected on PrEP, early HIV infection, early infection on PrEP, and late-stage infection. Male and female populations are modeled separately within each compartment. Only heterosexual transmission is modeled, with three distinct types of sexual relationships able to spread HIV: client and sex worker, marriage-like relationships, and nonpaid casual relationships. It is assumed that HIV transmission through the latter two relationships only occurs in the low-risk population. In other words, it is assumed that the only relationships engaged in by the high-risk population are client-sex worker relationships. Additionally, the model assumes that condoms are only used during client-sex worker relationships. A certain percentage of each risk group is assumed to move to the other group annually, at which point it is assumed they will discontinue PrEP, should they be in the PrEP group. In the event that a member of the PrEP group becomes infected, be it through failure of the treatment or lack of adherence, it is assumed that individuals will continue to take PrEP for an average of one year. Simulations suggested that PrEP would lead to a significant decrease in HIV infections in Africa. However, the study found that under certain circumstances PrEP could actually lead to an increase in HIV cases in southern India, primarily due to high rates of condom use in the sex industry. If the adoption of PrEP were to lead to a fairly small decrease in condom use, roughly 15%, the model predicts that the number of new HIV cases would increase. The authors assert that any implementation strategy must emphasize that PrEP is a supplement to condom use, not a substitution.

One concern raised with the introduction of PrEP therapy was its potential impact on the prevalence of drug resistance. Van de Vijver et al. performed a model comparison study in order to investigate this issue. (Van De Vijver et al., 2013) Three models of HIV transmission and disease progression were investigated. The first was the Synthesis Transmission Model, a stochastic model for heterosexual HIV transmission in sub-Saharan Africa beginning in the 1980s, with demographic information primarily incorporated from the HIV epidemic in South Africa. (Phillips et al., 2011) This model simulates individual-level HIV transmission based on age, gender, viral load, sexual risk behavior, presence of antiretroviral drugs, presence of specific drug-resistance mutations, and adherence to drug regimens. Sexual risk behavior was based on the number of short-term unprotected sex partners and presence of a long-term unprotected sex partner within a given three month period. In the adaptation by Van de Vijver et al, PrEP was introduced *via* a campaign targeting serodiscordant couples in long-term partnerships. Adherence was incorporated with both a fixed inherent tendency to adhere and a period-to-period variability in adherence. Adherence is further modified by drug toxicity, probability of a patient voluntarily interrupting clinic visits, and probability of interruptions in the drug supply. All of these parameters are assumed to vary by geographic region.

The second model in the comparison by Van de Vijver et al. is the South African Transmission Model, initially developed by Abbas et al. and based on PrEP trials in South and Sub-Saharan Africa. (Abbas et al., 2013) Like the previous model, it was calibrated based on the progression of the South African HIV-1 epidemic, and exclusively models heterosexual transmission. While the first model was entirely stochastic, this model provides a more deterministic framework by incorporating disease progression and viral dynamics. Briefly, the model stratifies the population based on gender, PrEP/ARV treatment status, infection status, stage of disease, and HIV-1 drug susceptibility, with susceptibility classified as either drug-sensitive or drug-resistant, and drug-resistance further classified as acquired or transmitted resistance. Inappropriate PrEP use, which is described as PrEP use subsequent to acute HIV infection, is modeled based on whether the individual taking PrEP is in a pre- or postseroconversion stage of the infection. After seroconversion it is assumed that PrEP use continues for a length of time corresponding to the HIV testing interval, the default being six months. In order to model sexual transmission, individuals of both genders are stratified into four sexual activity levels. These levels are used to construct a sexual activity matrix that describes, for any individual of gender *g* and activity level *k*, denoted *g_k_*, and a prospective partner of opposite gender *g’* and activity level *l*, denoted *g_l_’*, the probability of forming a sexual partnership denoted *g_kl_*. (Garnett and Anderson, 1993) The probability is derived from the total population of *g_l_’*, the tendency of *g_k_* to engage in assortative versus random mixing, and the rate at which *g_k_* individuals change partners when in a partnership with *g_l_’* individuals. The probability of HIV transmission for a single sex act within a sexual partnership is represented as a function of the partner’s ARV treatment status, disease stage, and HIV-1 variant. The total probability of HIV transmission for a partnership is then the per-sex act probability multiplied by the total number of sex acts for a partnership between two individuals *g_k_* and *g_l_’*. The protective effect of PrEP on an individual is modeled as a reduction in the susceptibility of that individual to the transmission of a given HIV variant, multiplied by the average adherence of the individual, which is itself determined by the individual’s adherence stratum.

The third and final model included in the comparison was the Macha Transmission Model. (Nichols et al., 2013) While the other studies included in the comparison focused on the South African HIV epidemic, the model’s namesake is a rural hospital in Southern Zambia, roughly 80 kilometers from the nearest town, and serves as the only major HIV clinic for roughly 90,000 people. Despite being calibrated to a different population, the Macha model shares a number of features with the South African model. The Macha model is a deterministic, compartmental model incorporating HIV disease progression. Once again the population is stratified based on sexual activity level, with higher activity levels corresponding to a greater number of sexual partners per year. The disease progression model depicts the stages of infection as acute HIV, chronic HIV, early AIDS, and late AIDS, with the AIDS compartment subdivided primarily to reflect changes in sexual activity associated with progression to AIDS, assuming that early stage AIDS is characterized by a reduction in sexual activity, and therefore transmission, while sexual activity halts entirely in the late stage of the disease. Just as in the South African model, the Macha model adapts the mixing matrix described by Garnett and Anderson in order to model transmission in a heterosexual population stratified by sexual activity level. (Garnett and Anderson, 1993) However, the Macha model differs from the South African model in that it stratifies the infected population into individuals who have undergone HIV testing and are aware of their infection, and those who are unaware. The model assumes that individuals who are aware of their seropositive status may make some effort to reduce their acquisition rate of new sexual partners. It assumes this effect is not uniform across all sexual activity levels, with the two lowest levels reducing acquisition rates by up to 40% while the two highest levels show no change in behavior. This stratification leads to two mixing matrices; one is identical to the previously described matrix and applies to individuals who are unaware of their HIV infections, while a second matrix incorporates the reduction in the rate of partner acquisition for individuals who are aware of their infection.

## Conclusion

PrEP therapy for HIV remains an active and growing field of research. In addition to the currently approved PrEP therapies, several alternatives are in the mid to late stages of development. Many of these therapies are long-acting or on-demand approaches that aim to address problems of adherence and availability. The primary aim of this review was to provide an overview of the available pharmacokinetic models of both current PrEP regimens and antiretrovirals currently under investigation as PrEP agents, while highlighting some of the challenges associated with modeling more complex formulations and delivery systems. In addition, it is important to note the challenges involved in translating *in vitro* and ex vivo estimates of antiretroviral efficacy into estimates of clinical outcomes. Finally, an overview of some of the disease progression and viral transmission models that have been used to investigate HIV PrEP has been included, as population-level variables such as the frequency and routes of HIV exposure, propensity to modify high-risk behavior, and crucially, patient adherence to PrEP regimens, must be taken into account when modeling HIV PrEP at the population level. The diverse array of administration routes, compounds and dosing regimens presents novel challenges to drug development. In silico modeling and simulation approaches offer powerful tools to inform clinical trials, and allow for rapid investigation of pharmacokinetic and pharmacodynamic questions that arise during the drug development process. Moreover, modeling and simulation approaches provide investigators with the ability to examine scenarios related to changes in transmission, treatment adherence, and sexual behavior that might otherwise be precluded from clinical studies due to practical or ethical concerns. Ultimately, there are still many aspects of the HIV PrEP problem space that have yet to be explored through computational modeling.

## Author Contributions

TS drafted the review article. TS, RB, and KK revised and edited the article for clarity and content.

## Funding

RB is the recipient of grant funding from the US National Institutes of Health grant 1U19AI120249.

## Conflict of Interest

RB has received consulting fees from Janssen Pharmaceuticals and grant funding from Takeda Pharmaceuticals, the US Department of Defense, and the US National Institutes of Health. KK was employed by Metrum Research Group.

The remaining author declares that the research was conducted in the absence of any commercial or financial relationships that could be construed as a potential conflict of interest.
