# Supplementary material for: Modeling HIV Pre-Exposure Prophylaxis
Source: Front Pharmacol. 2020 Jan 31;10:1514. doi: 10.3389/fphar.2019.01514 (PMC7005100; doi:10.3389/fphar.2019.01514)
Supplement: Supplementary file 1 [file DataSheet_1.docx]

Supplementary Material

## Supplementary Figures


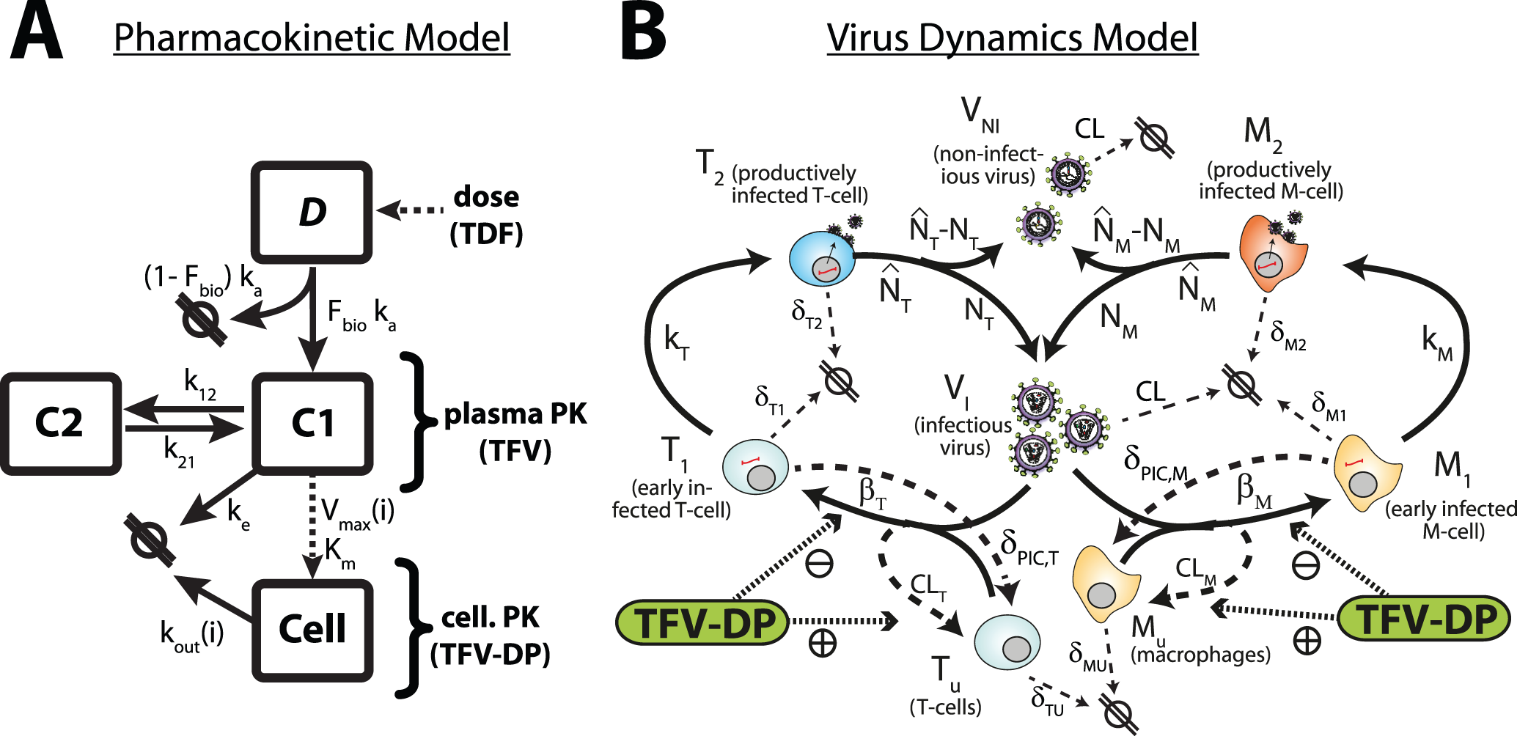


**Supplementary Figure 1.** Pharmacokinetic model for tenofovir disoproxil (TDF), tenofovir (TFV), and tenofovir diphosphate (TFV-DP). The dose is administered as TDF, and is rapidly to converted to TFV upon absorption into plasma. Circulating TFV is taken up into cells and phosphorylated to TFV-DP via a saturable process with Michaelis-Menten kinetics. Abbreviations: k_a_ – absorption rate constant, F_bio_ – bioavailability, k_12_/k_21_ – distribution rate constants between plasma compartments 1 and 2, k_e_ – plasma elimination rate constant, V_max_ – maximum conversion rate of TFV to TFV-DP, K_m_ – TFV concentration corresponding to half-maximal conversion rate, k_out_ – intracellular elimination rate constant. Reproduced from (Duwal et al., 2012){Duwal, 2012 #187} under the terms of the Creative Commons Attribution License.


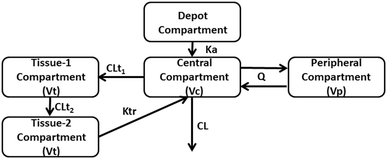


Supplementary Figure 2. Compartmental model for tenofovir pharmacokinetics in plasma and rectal tissue. Tenofovir disposition is described using a two-compartment model for plasma PK. Rectal tissue PK was modeled as two separate tissue compartments, where tissue concentrations were sampled from the first compartment. The second tissue compartment functions as a delay compartment for transfer of TFV from tissue to plasma, and may represent metabolite formation, degradation and recycling. Abbreviations: Ka – absorption rate constant, CL – systemic clearance, Q – intercompartmental clearance, CLt_1_ – clearance from central compartment to tissue compartment 1, CLt_2_ clearance from tissue compartment 1 to tissue compartment 2, K_tr_ – rate constant for transit from tissue compartment 2 to central compartment, V_c_ – central compartment volume, V_p_ – peripheral compartment volume, V_t_ – tissue compartment volume Reproduced from (Collins et al., 2017) with permission.


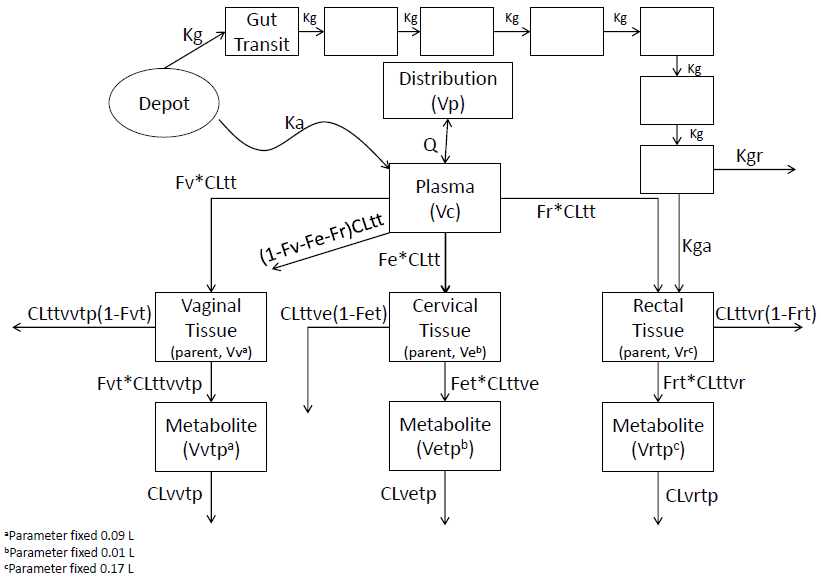


Supplementary Figure 3. Semi-physiologic model incorporating parent and metabolite compartments in vaginal, cervical and colorectal tissue. Tissue compartment volumes were fixed based on estimated physiological volumes. The model assumes linear kinetics and estimates separate rate constants for parent and metabolite formation and clearance within each tissue type. Abbreviations: K_g_ – rate constant for gut transit compartments modeling delay in oral dose reaching rectal tissue, K_gr_ – rate constant for clearance from gut of unabsorbed drug, K_ga_ – rate constant for absorption of drug into rectal tissue, Ka – rate constant for drug entering systemic circulation, CL_tt_ – total clearance to vaginal, cervical and rectal tissues, F_v_: fraction of tissue clearance to vaginal tissue, F_e_ – fraction of tissue clearance to cervical tissue, F_r_ – fraction of tissue clearance to rectal tissue, CL_ttvvtp_ – metabolic clearance of drug from vaginal tissue compartment, CL_ttve_ – metabolic clearance of drug from cervical tissue compartment, CL_ttvr_ – metabolic clearance of drug from rectal tissue compartment, CL_vvtp_ – clearance of metabolite from vaginal tissue compartment, CL_vetp_ – clearance of metabolite from cervical tissue compartment, CL_vrtp_ – clearance of metabolite from rectal tissue compartment, V_c_: central compartment volume of distribution, V_p_: peripheral compartment volume of distribution, Q: intercompartmental clearance, Reproduced from (Cottrell et al., 2016) with permission.


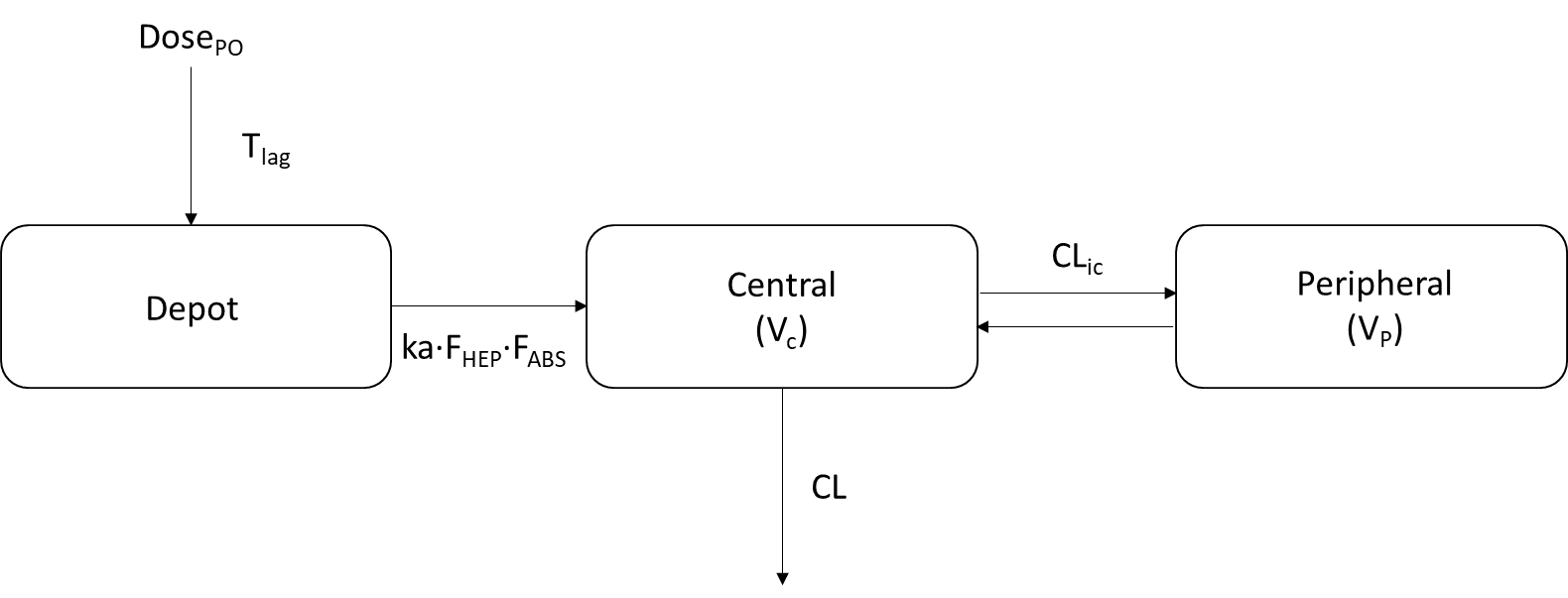


Supplementary Figure 4. Basic model for orally administered maraviroc (MVC). MVC disposition is assumed to be linear, with the exception of the fraction absorbed from the gut, which was modeled using a sigmoidal relationship between dose and fraction absorbed. ABS_Emax_ is fixed to 1, representing the assumption that an infinitely high dose would be completely absorbed. Abbreviations: T_lag_ lag time for absorption, ka: first-order absorption rate constant, F_HEP_: fraction of dose escaping hepatic first-pass metabolism, F_ABS_: fraction of dose absorbed from gut, CL_ic_: intercompartmental clearance, CL: systemic clearance, V_c_: volume of central compartment, V_p_: volume of peripheral compartment. Figure created based on description of model structure in (Chan et al., 2008), adapted with permission.


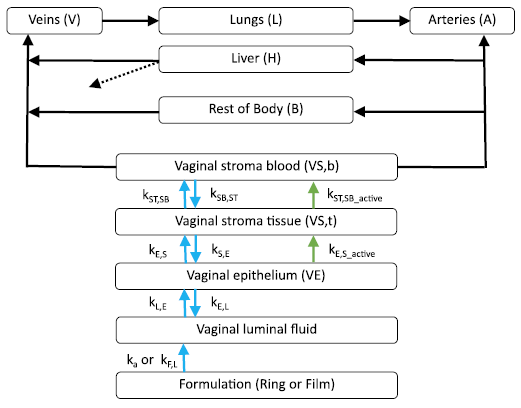


Supplementary Figure 5. Physiologically-based PK mode for vaginal delivery of Dapirivine (DPV). Drug is released into the vaginal luminal fluid using to a formulation-specific process. From luminal fluid, first order rate constants describe the movement of drug between the luminal fluid, epithelium, tissue stroma, and vaginal stromal blood. Active transport is incorporated for uptake of the ionized form of the drug from epithelium to stroma and stroma to blood Abbreviations: k_a_/k_F,L_– rate constants for release of drug from ring or film formulation to vaginal luminal fluid, k_L,E_ –.rate consant for absorption from vaginal luminal fluid to vaginal epithelium, k_E,L_ – rate constant for efflux of drug from vaginal epithelium to vaginal luminal fluid, k_E,S_ – rate constant for passive movement of drug from vaginal epithelium to vaginal stroma, k_S,E_ – rate constant for passive movement of drug from vaginal stroma to vaginal epithelium, k_E,S­active_ – rate constant for active transport of drug from vaginal epithelium to vaginal stroma, k_ST,SB_ – rate constant for passive movement of drug from vaginal stroma to vaginal stroma blood, k_SB,ST_ – rate constant for passive movement vaginal stroma blood to vaginal stroma, k_ST,SB_active_ – rate constant for active transport of drug from vaginal stroma to vaginal stroma blood, V_E_ – volume of vaginal epithelium, V_S,t_ – volume of vaginal sromal tissue, V_S,b_ – volume of vaginal stromal blood, A – arterial blood volume, V –venous blood volume, L – lung volume, H – liver volume, B – volume of bodily tissues not explicitly specified in model. Reproduced from (Kay et al., 2018) under the terms of the Creative Commons Attribution License.

## Supplementary Tables

| **Single Dose Administration, Day 1** | | | | | | | |
| --- | --- | --- | --- | --- | --- | --- | --- |
| **FTC Dose** | **Statistic** | **C_max_ (µg/mL)** | **t_max_ (h)** | **AUC_0-t_ (µg·h/mL)** | **AUC_0-∞_ (µg·h/mL)** | **t_1/2_ (h)** | **CL/F (mL/min)** |
| **25 mg BID (n = 9)** | Mean CV% | 0.15 24 | 1.60 53 | 0.60 21 | 0.68 21 | 3.61 21 | 638 22 |
| **100 mg BID (n = 8)** | Mean CV% | 0.90 37 | 2.41 52 | 3.68 27 | 4.14 35 | 3.26 23 | 440 29 |
| **200 mg BID (n = 8)** | Mean CV% | 1.63 21 | 1.14 34 | 5.99 16 | 6.37 16 | 2.83 10 | 535 15 |
| **100 mg QD (n = 8)** | Mean CV% | 0.98 43 | 1.54 45 | 3.16 33 | 3.42 32 | 3.48 24 | 537 35 |
| **200 mg QD (n = 8)** | Mean CV% | 1.54 38 | 2.25 46 | 6.47 18 | 7.07 19 | 2.98 14 | 489 22 |
| **100 mg combined (n = 16)** | Mean CV% | 0.94 40 | 1.97 55 | 3.42 30 | 3.78 34 | 3.37 23 | 489 34 |
| **200 mg combined (n = 16)** | Mean CV% | 1.59 30 | 1.70 56 | 6.23 17 | 6.72 18 | 2.90 12 | 512 18 |
| **Steady-State Administration, Day 10** | | | | | | | |
| **FTC Dose** | **Statistic** | **C_max,ss_ (µg/mL)** | **t_max,ss_ (h)** | **C_min,ss_ (µg/mL)** | **AUC_τ_ (µg·h/mL)** | **t_1/2_ (h)** | **CL_ss_/F (mL/min)** |
| **25 mg BID (n = 9)** | Mean CV% | 0.23 30 | 1.39 39 | 0.029 32 | 0.99 17 | 5.26 8 | 430 17 |
| **100 mg BID (n = 8)** | Mean CV% | 1.15 19 | 1.69 63 | 0.148 68 | 5.39 35 | 4.25 15 | 339 30 |
| **200 mg BID (n = 8)** | Mean CV% | 2.05 34 | 1.23 28 | 0.171 23 | 8.47 26 | 3.59 16 | 415 26 |
| **100 mg QD (n = 8)** | Mean CV% | 0.90 20 | 1.21 23 | 0.035 50 | 4.10 22 | 9.49 21 | 423 21 |
| **200 mg QD (n = 8)** | Mean CV% | 1.72 53 | 2.00 48 | 0.047 24 | 8.00 15 | 8.24 31 | 425 15 |

Supplementary Table 1. Summary of PK parameters for emtricitabine (FTC) in HIV-1 infected patients. Values are presented as arithmetic mean and coefficient of variability. Reproduced from (U.S. Food and Drug Administration, 2003).

| **MVC Dose** | **Day (n)** | **AUC_τ_  (ng mL^-1^ h)** | **AUC (ng mL^-1^ h)** | **C_max_ (ng mL^-1^)** | **T_max_ (h)** | **t_1/2_ (h)** |
| --- | --- | --- | --- | --- | --- | --- |
|  |  | **Mean (CV%)** | **Mean (CV%)** | **Mean (CV%)** | **Mean (SD)** | **Mean (SD)** |
| **3 mg BID** | 1 (5) | NC | NC | 0.67 (52) | 0.9 (0.4) | NC |
|  | 7 (5) | 6.6 (32) | NC | 1.32 (31) | 1.1 (0.2) | NC |
|  | 12 (4) | 4.2 (69) | NC | 0.83 (35) | 0.6 (0.3) | NC |
| **10 mg BID** | 1 (5) | 11.8 (33) | NC | 2.26 (37) | 1.8 (1.4) | NC |
|  | 7 (5) | 19.0 (25) | NC | 2.71 (35) | 1.9 (2.3) | NC |
|  | 12 (5) | 22.2 (24) | 44.8 (8.6) | 3.33 (41) | 1.3 (1.5) | 15.2 (3.1) |
| **25 mg BID** | 1 (9) | 46.1 (51) | 74.6 (50) | 8.72 (62) | 3.3 (2.3) | 10.8 (2.7) |
|  | 7 (8) | 92.0 (47) | NC | 18.6 (51) | 3.1 (1.7) | NC |
|  | 12 (8) | 98.6 (49) | 236 (35) | 16.2 (48) | 3.3 (2.1) | 13.9 (2.2) |
| **100 mg BID** | 1 (9) | 512 (21) | 579 (21) | 187 (43) | 2.2 (0.9) | 7.76 (0.57) |
|  | 7 (9) | 636 (26) | NC | 159 (42) | 2.5 (1.4) | NC |
|  | 12 (9) | 686 (24) | 1018 (23) | 181 (30) | 2.5 (1.9) | 18.5 (2.5) |
| **300 mg BID** | 1 (9) | 2157 (47) | 2422 (46) | 538 (46) | 1.6 (1.4) | 8.63 (2.2) |
|  | 7 (9) | 2641 (43) | NC | 674 (47) | 1.5 (1.3) | NC |
|  | 12 (9) | 3609 (32) | 4561 (31) | 854 (32) | 2.6 (1.5) | 16.4 (2.3) |
| **600 mg QD (cohort 3)** | 1 (9) | 5877 (20) | 6074 (20) | 1317 (38) | 3.3 (0.9) | 7.74 (0.99) |
|  | 7 (9) | 6982 (27) | 7650 (26) | 1351 (38) | 2.6 (1.3) | 15.3 |
|  | 12 (0) | NM | NM | NM | NM | NM |
| **600 mg QD (cohort 5)** | 1 (9) | 5545 (23) | 5717 (22) | 1322 (41) | 2.1 (1.4) | 7.84 (1.4) |
|  | 7 (9) | NC | NC | 1204 (45) | 2.8 (1.8) | NC |
|  | 12 (9) | 6440 (12) | 7177 (13) | 1361 (20) | 2.3 (1.1) | 17.2 (3.9) |

Supplementary Table 2. Mean maraviroc (MVC) pharmacokinetics for 12-day multiple dose study. Geometric means were used for AUC_τ_, AUC, and C_max_. Arithmetic means were used for T_max_ and t_1/2_. NC: not calculated. NM: not measured. All doses were administered as oral solutions, except for cohort 3 and cohort 5, which received oral tablets. Reproduced from (Abel et al., 2008) with permission.

# References

Abel, S., Van Der Ryst, E., Rosario, M.C., Ridgway, C.E., Medhurst, C.G., Taylor-Worth, R.J., et al. (2008). Assessment of the pharmacokinetics, safety and tolerability of maraviroc, a novel CCR5 antagonist, in healthy volunteers. *British Journal of Clinical Pharmacology* 65(1)**,** 5-18. doi: 10.1111/j.1365-2125.2008.03130.x.

Chan, P.L.S., Weatherley, B., and McFadyen, L. (2008). A population pharmacokinetic meta-analysis of maraviroc in healthy volunteers and asymptomatic HIV-infected subjects. *British Journal of Clinical Pharmacology* 65(1)**,** 76-85. doi: 10.1111/j.1365-2125.2008.03139.x.

Collins, J.W., Heyward Hull, J., and Dumond, J.B. (2017). Comparison of tenofovir plasma and tissue exposure using a population pharmacokinetic model and bootstrap: a simulation study from observed data. *Journal of Pharmacokinetics and Pharmacodynamics* 44(6)**,** 631-640. doi: 10.1007/s10928-017-9554-9.

Cottrell, M.L., Yang, K.H., Prince, H.M.A., Sykes, C., White, N., Malone, S., et al. (2016). A Translational Pharmacology Approach to Predicting Outcomes of Preexposure Prophylaxis Against HIV in Men and Women Using Tenofovir Disoproxil Fumarate with or Without Emtricitabine. *Journal of Infectious Diseases* 214(1)**,** 55-64. doi: 10.1093/infdis/jiw077.

Duwal, S., Schütte, C., and von Kleist, M. (2012). Pharmacokinetics and pharmacodynamics of the reverse transcriptase inhibitor tenofovir and prophylactic efficacy against HIV-1 infection. *PLoS ONE* 7(7)**,** e40382. doi: 10.1371/journal.pone.0040382.

Kay, K., Shah, D.K., Rohan, L., and Bies, R. (2018). Physiologically‐based pharmacokinetic model of vaginally administered dapivirine ring and film formulations. *British Journal of Clinical Pharmacology* 84(9)**,** 1950-1969. doi: 10.1111/bcp.13625.

U.S. Food and Drug Administration. 2003. NDA 21500 Clinical Pharmacology and Biopharmaceutics Review. Available: <https://www.accessdata.fda.gov/drugsatfda_docs/nda/2003/021500_emtriva_toc.cfm>.
